# Positive Selection Shaped the Convergent Evolution of Independently Expanded Kallikrein Subfamilies Expressed in Mouse and Rat Saliva Proteomes

**DOI:** 10.1371/journal.pone.0020979

**Published:** 2011-06-14

**Authors:** Robert C. Karn, Christina M. Laukaitis

**Affiliations:** Department of Medicine, College of Medicine, University of Arizona, Tucson, Arizona, United States of America; University of Cambridge, United Kingdom

## Abstract

We performed proteomics studies of salivas from the genome mouse (C57BL/6 strain) and the genome rat (BN/SsNHsd/Mcwi strain). Our goal was to identify salivary proteins with one or more of three characteristics that may indicate that they have been involved in adaptation: 1) rapid expansion of their gene families; 2) footprints of positive selection; and/or 3) sex-limited expression. The results of our proteomics studies allow direct comparison of the proteins expressed and their levels between the sexes of the two rodent species. Twelve members of the *Mus musculus* species-specific kallikrein subfamily Klk1b showed sex-limited expression in the mouse saliva proteomes. By contrast, we did not find any of the *Rattus norvegicus* species-specific kallikrein subfamily Klk1c proteins in male or female genome rat, nor transcripts in their submandibular glands. On the other hand, we detected expression of this family as transcripts in the submandibular glands of both sexes of Sprague-Dawley rats. Using the CODEML program in the PAML package, we demonstrate that the two rodent kallikrein subfamilies have apparently evolved rapidly under the influence of positive selection that continually remodeled the amino acid sites on the same face in the members of the subfamilies. Thus, although their kallikrein subfamily expansions were independent, this evolutionary pattern has occurred in parallel in the two rodent species, suggesting a form of convergent evolution at the molecular level. On the basis of this new data, we suggest that the previous speculative function of the species-specific rodent kallikreins as important solely in wound healing in males be investigated further. In addition to or instead of that function, we propose that their sex-limited expression, coupled with their rapid evolution may be clues to an as-yet-undetermined interaction between the sexes.

## Introduction

Over the past ten years, numerous genome sequences have appeared and comparative studies of these datasets have enhanced our understanding of gene evolution. For example, studies of mammalian genomes suggest that the vast majority of genes have strongly conserved coding sequences, and generally occur only in single copies [1], [2], [3]. By contrast, genes involved in adaptation and functional innovation often show the footprints of positive selection in elevated ratios of nonsynonymous to synonymous nucleotide substitutions (*dN/dS*; sometimes reported as the rate *Ka/Ks*; [4]) in their coding regions [5], [6], [7], [8], [9]. Moreover, they are subject to frequent duplication, deletion and pseudogene formation [10], [11]. Prevalent among rapidly evolving genes are those involved in immunity, reproduction, chemosensation and toxin metabolism [10].

A high rate of amino acid substitution is a possible signature of adaptive evolution. Proteins involved in reproductive fitness have evolved unusually rapidly across diverse groups of organisms [10], [12], [13], [14]. In the case of reproductive proteins, coevolutionary cycles involving adaptation and counter adaptation are expected to apply continuous selective pressure, resulting in rapid changes at amino acid sites involved in the function of the protein. These proteins often have roles in sperm competition, host immunity to pathogens, and manipulation of female reproductive physiology and behavior; however, in many other cases, the function of the rapidly evolving protein is unknown.

In addition to showing higher rates of evolution and higher rates of duplication and loss, candidate reproductive proteins also often show sex-limited expression. This is obvious in those genes whose protein products are synthesized in organs and glands found in only one of the two sexes, apparently because they confer a reproductive advantage in fertility and/or gamete recognition [15], [16]. On the other hand, one seldom associates sex-limited expression with exocrine glands such as the salivary glands. However, some species such as the house mouse (*Mus musculus*) and some strains of rats (*Rattus norvegicus*) show impressive elaboration of a specific tissue of the submandibular gland, the granulated convoluted tubular (GCT) tissue, often only in males following puberty [17]. One consequence of this sex-limited expression is that submandibular glands which have elaborated GCT begin to produce serine proteases called kallikreins encoded in subfamilies that have recently expanded independently in house mice and rats [18]. The two expansions produced analogous clusters of potentially expressed paralogs (note that the rat subfamily pseudogenes are actually paralogs of *Klk2ps*) insofar as they both expanded from the basal *Klk1* paralog [19]. The actual function of this sex-limited kallikrein expression is unknown, although there has been unsupported speculation that they promote wound healing in males injured in male-male aggression during the course of reproduction [20].

Three families of putative proteinaceous pheromones studied in the house mouse fit into the reproduction category mentioned above: the androgen-binding proteins (ABPs), exocrine gland–secreting peptides (ESPs), and major urinary proteins (MUPs). Each of these pheromones is thought to communicate different information and their gene families show different degrees of volatility in terms of duplications and deletions identified with the Mouse Paralogy Browser [11]. We are interested in identifying other rapidly evolving genes encoding proteins secreted into saliva because it is possible that there are other salivary proteins with roles in reproduction that have yet to be described. Our goal was to identify salivary proteins with one or more of the three characteristics described above: rapid expansion of their gene families; footprints of positive selection; and/or sex-limited expression. To accomplish this, we employed multidimensional protein identification technology (MUDPIT) to produce saliva proteomes of the genome mouse (C57BL/6 strain) and the genome rat (BN/SsNHsd/Mcwi strain) and compared their proteins. A human salivary proteome has been produced by a similar method to that we report here [21] and, in addition, the UCLA Human Saliva Proteome Project (HSPP) is available on-line at the Salivaomics Knowledge Database (SKB) (http://www.skb.ucla.edu/). No similar resource is available for rodent saliva proteomes although a brief description of a shotgun proteomic method using two-dimensional nano-flow LC tandem mass spectrometry to study rat salivas appeared recently [22].

Both the mouse and rat species-specific kallikrein families fit several of the criteria we sought: they are recently expanded gene families and, at least in mouse saliva, show impressive sex-limited expression. We report here the nearly absolute sex-limited expression of the Klk1b subfamily of kallikreins in the genome mouse, in general agreement with the observations of others using more limited lines of investigation (see below). Only one member of that family was expressed in both sexes, Klk1b5. Surprisingly, the genome rat did not express any of the nine Klk1c subfamily of kallikreins in either sex, only the basal Klk1 and that in both sexes. Since others had observed expression of all of the Klk1c subfamily kallikrein transcripts in the submandibular glands of male Sprague-Dawley rats [23], we replicated their analysis and extended it to include submandibular glands of female Sprague-Dawley rats. We observed expression of all the Klk1c subfamily kallikrein transcripts in both sexes of Sprague-Dawley rats and we support this with histological analyses showing that GCT elaboration is similar between male and female rats, but varies between strains. Thus the expression of the two species-specific subfamilies of kallikreins in mouse and rat differs significantly, with the expression in rat being dependent on the strain studied and independent of gender.

In addition to studying the expression of the two subfamilies of rodent kallikreins, we performed an evolutionary analysis on the paralogs within each rodent kallikrein subfamily. This included revisiting the question of concerted evolution [24] and studying the role of positive selection using the CODEML program of the phylogenetic analysis by maximum likelihood (PAML) package [25], [26]. From the comparison, we conclude that these two rodent subfamilies evolved in a very similar way that constantly remodeled the same face on the exterior of the protein in each species, suggesting that these two subfamilies have experienced convergent evolution. We question the earlier proposal that the function of these sex-limited salivary kallikrein expressions involves wound healing only in males and we reconsider it in light of the results we report here. In addition to or instead of that function, we propose that their sex-limited expression, coupled with their rapid evolution may be clues to an as-yet-undetermined interaction between the sexes, perhaps one involving inactivation and/or damaging proteins with a signaling function produced by competing animals.

## Materials and Methods

### Saliva sources and treatment

All animal manipulation was performed in accordance with University of Arizona IACUC procedures under protocols 08-138 (mice) and 10-158 (rats). Male and female mice of strain C57BL/6 were obtained from Jackson Laboratory (Bar Harbor, Maine) and fed Harlan-Teklad #7013 chow. Male and female rats of strain BN/SsNHsd/Mcwi were obtained from the Medical College of Wisconsin (Milwaukee) and of the Sprague-Dawley strain were obtained from Harlan. The rats were fed the Harlan Teklad #2018 chow. Salivas were collected using isoproterenol stimulation as described previously [27]. The salivas were dialyzed against 100 mM NH_4_HCO_3_ buffer, pH 8.3 and provided to the Proteomics Core Facility of the University of Arizona. The Proteomics Core personnel quantitated the protein in the samples, digested 30 ug of each with trypsin and analyzed the samples with MUDPIT proteomics technology as described below.

### Multidimensional Chromatography Coupled to Tandem Mass Spectrometry (LC-LC-MS/MS)

Digested peptide mixtures were loaded by pressure packing onto a 100 micron fused silica capillary column packed with 3.5 cm of 5-micron beads coated with PolySulfoethyl-Asp strong cation exchanger (SCX, PolyLC Inc., Columbia, MD). This SCX material was packed against a microfilter assembly (Upchurch Scientific, Oak Harbor, WA). The microfilter assembly was then connected to a 100 micron id fused silica capillary column that was previously packed with 8 cm of 5 micron Zorbax Eclipse XDB-C18 packing material (Agilent, Santa Clara, CA) that was also the nanospray emitter tip made by pulling the fused silica to a 5 micron tip using a laser puller (Sutter Instrument, Novato, CA). This dual column was placed in-line with a microbore HPLC system (Surveyor; Thermo Electron, San Jose, CA, USA) that was modified to operate at capillary flow rates using a simple T-piece flow-splitter. Peptides were eluted in a gradient using Buffer A (0.1% formic acid), Buffer B (acetonitrile/0.1% formic acid), Buffer C (250 mM ammonium acetate), and Buffer D (1.5 M ammonium acetate) at a flow rate of 400 nL/min. Twelve steps were then performed: (step 1) 0% C with a gradient of 5–50% B over 90 min followed by a column clean-up of 5 min. 50–98% B and an equilibration of 20 min. 5% B; (steps 2–11) X% C (where X = 10–100% C increased in increments of 10) loaded over 4 min and then washed with 5% B for 7 min followed by a gradient of 5–50% B over 60 min. Each gradient was followed by a column clean-up of 5 min. 50–98% B and an equilibration of 20 min. 5% B; (step 12) 50% D loaded over 4 min and then washed with 5% B for 7 min followed by a gradient of 5–50% B over 60 min. The flow rate was 1.0 uL/min for the 7 min wash following each salt fraction and for each final 5% B equilibration. The HPLC column eluent was directed into the ESI source of an LTQ linear ion trap mass spectrometer (Thermo Fisher Scientific). Electrospray voltage of 2.0 kV was applied using a gold electrode via a liquid junction up-stream of the column. Spectra were scanned over the range 400–1500 amu. Automated peak recognition, dynamic exclusion (exclude after 3 acquisitions in 40 seconds and then exclude for 3 minutes), and daughter ion scanning of the top seven most intense ions were performed using the Xcalibur data system (Thermo Fisher Scientific).

### Criteria for protein identification

Scaffold (version 3.0; Proteome Software Inc., Portland, OR) was used to validate MS/MS based peptide and protein identifications. Peptide identifications were accepted if they exceeded specific database search engine thresholds. Sequest identifications required at least deltaCn scores of greater than 0.08 and XCorr scores of greater than 1.8, 2.5, 3.5 for singly, doubly, triply charged peptides. X! Tandem identifications required at least −Log (Expect Scores) scores of greater than 3.0. Protein identifications were accepted if they contained at least 2 identified peptides. Proteins that contained similar peptides and could not be differentiated based on MS/MS analysis alone were grouped to satisfy the principles of parsimony. The IPI database searches were augmented with FASTA lists of all *Abp* paralogs both in the case of mouse (30 *Abpa* paralogs and 34 *Abpbg* paralogs) and rat (three of each).

### Polymerase chain reaction (PCR) identification of rat kallikrein transcripts

Male and female rats of the Sprague-Dawley strain at 75 days of age were obtained from Harlan (Placentia, CA). Other strains were obtained as described above. Rats were sacrificed by CO_2_ inhalation and their salivary glands removed. We isolated total RNA from a portion of one of the submandibular glands of male and female BN/SsNHsd/Mcwi and Sprague-Dawley rats as described previously [28]. We produced cDNA by reverse transcription of equivalent amounts of RNA template using a Fermentas (Glen Burnie, MD) first-strand cDNA kit and an oligo dT primer. Expression of *Klk1c* genes was assayed using this cDNA as a template and primers of the sequences designed by [23] obtained from Bioneer Inc. (Alameda, CA). Before using the primers to amplify cDNAs, they were tested on Sprague-Dawley rat genomic DNA and all primer sets successfully amplified DNA the same sizes as those predicted and identified by virtual PCR with the primer sequences in the UCSC genome browser [29]. The PCR products were analyzed by agarose gel electrophoresis as described previously [28].

### Histology of rat submandibular glands

The second submandibular gland isolated from each rat was fixed in 10% neutral buffered formalin for 24 hours before embedding in paraffin. Serial sections of 4.5 µ were cut and stained with hematoxylin and eosin (University of Arizona Health Sciences Center Cell Biology and Anatomy Histology Service Lab). Representative electronic images were collected at 10× and 20× magnifications using a light microscope.

### Detecting Gene Conversion

Sequences were aligned with CLUSTALW in the BioEdit package (http://www.mbio.ncsu.edu/bioedit/bioedit.html). The program GENECONV (http://www.math.wustl.edu/~sawyer/geneconv/gconvdoc.pdf) was used to search for gene conversion tracks. GENECONV seeks aligned DNA or protein segments for which a pair of sequences is sufficiently similar to suggest that gene conversion occurred. These are classified as inner or outer fragments. Inner fragments are evidence of a possible gene conversion event between ancestors of two sequences in the alignment. Outer fragments are runs of unique sites that may be evidence of past gene conversion events that originated from outside of the alignment or else from within the alignment but such that evidence of the source has been destroyed by later mutation or gene conversion. GC content of the mouse and rat kallikrein subfamily regions was determined using an online calculator provided by EnCore Biotechnology, Inc. (http://www.encorbio.com/protocols/Nuc-MW.htm).

### Data analysis

Mouse and rat subfamily kallikrein amino acid sequences were obtained from NCBI using the accession numbers reported in [30]. They were modified to contain only the cleaved pro-kallikrein sequences representing active enzymes. These were aligned using CLUSTALX [31], [32]. Phylogenetic trees were constructed from the alignments using the program PAUP* [33] and these were displayed in TreeView [34]. In PAUP*, neighbor-joining (NJ) distance parameters with Jukes-Cantor correction and random-seeding were used to calculate divergences between rodent sequences and to create trees with proportional branch lengths and bootstrap values were calculated with 1000 replications. The mouse *Klk1b* tree obtained with the parsimony criterion had the same topology and the rat *Klk1c* parsimony tree had essentially the same topology as those obtained with NJ (not shown). Positive selection was assessed in the program CODEML in the PAML 3.14 package [25], [26]. For each gene, three different comparisons of neutral and selection models gave similar results (M1 vs. M2, M7 vs. M8, and M8A vs. M8 [8], [35], [36]). Model M1 (neutral) allows two classes of codons, one with *dN/dS* over the interval (0,1) and the other with a *dN/dS* value of one. Model M2 (selection) is similar to M1 except that it allows an additional class of codons with a freely estimated *dN/dS* value. Model M7 (neutral) estimates *dN/dS* with a beta-distribution over the interval (0, 1), whereas model M8 (selection) adds parameters to M7 for an additional class of codons with a freely estimated *dN/dS* value. M8A (neutral) is a special case of M8 that fixes the additional codon class at a *dN/dS* value of one.

The three-dimensional structures of mouse Klk1 and rat Klk1 were modeled using the PHYRE 0.2 threading program (http://www.sbg.bio.ic.ac.uk/phyre/), and the resulting models were visualized using PYMOL (open-source 1.2.8; http://www.pymol.org/). Sites under positive selection were mapped onto the structural models using PYMOL and incorporated into a figure.

## Results

### Mouse and rat saliva proteomes

MUDPIT analyses involve two different chromatographic separations coupled to tandem mass spectrometry (LC-LC-MS/MS) to produce spectra that can be used to identify peptides corresponding to known protein sequences. We performed this type of analysis on isoproterenol-stimulated salivas from three males and three females of the genome mouse (C57BL/6 strain). Our primary goal was to identify salivary proteins that have been involved in adaptation because they are members of large families and show positive selection and/or sex-limited expression. To provide a broader context to the project, we also performed MUDPIT analyses on the salivas of one male and one female genome rat (BN/SsNHsd/Mcwi strain). We chose the genome strains so that we and other researchers could make direct comparisons of the proteins in these saliva proteomes to the genes in the sequenced genomes.

The variety of proteins ascertained in the MUDPIT analyses is a function of the specific database search engine thresholds set in the Scaffold program. Figure S1 shows a scatterplot of identifications made in mouse saliva by the Sequest algorithm vs. those made by the X! Tandem algorithm with the custom criteria specified in Methods for the Scaffold software. Figure S1 shows that, as the settings become more stringent, an increasing number of proteins are filtered out of the results and the cohort that remains has a yet higher overall probability of containing valid identifications. Using the criteria specified in Methods, we report 81 proteins identified in mouse saliva (Tables S1, S2, S3) and 46 proteins in rat saliva (Tables S4 and S5). Fifteen of these proteins were found in the salivas of both rodent species (19% of the mouse proteins and 33% of the rat proteins; Table 1), including proteins such as amylase, parotid secretory protein and cysteine-rich secretory protein previously identified in other studies. Because both the mouse and rat saliva proteomes contained putative uncharacterized proteins, we obtained the sequences of those from the IPI database and then used the PSI-Blast program on the National Center for Biotechnology Information (NCBI; http://www.ncbi.nlm.nih.gov/) website to obtain the most closely related sequence in the other rodent. This resulted in the identification of only one additional shared protein, lactoperoxidase (Table 1).

**Table 1:** Proteins common to both mouse and rat saliva proteomes.

	IPI Database number		
Protein name	*Mus musculus*	*Rattus norvegicus*	Notes
Acidic mammalian chitinase	IPI00346516	IPI00421295	
Alpha-amylase 1	IPI00315893	IPI00198466	
Carbonic anhydrase 6	IPI00114881	IPI00569317	
Cysteine-rich secretory protein 1	IPI00132195	IPI00193789	
Deoxyribonuclease-1	IPI00308684	IPI00230876	
Kallikrein-1	IPI00323869	IPI00231193	Rat “Klk1b3” in the IPI database but aka Klk1 (orthologous to mouse Klk1)
Lactoperoxidase	IPI00133770	IPI00370706	Rat “Putative uncharacterized protein Lpo”
Long palate, lung and nasal epithelium carcinoma-associated protein 1	IPI00117756	IPI00780659	
Nucleobindin-2	IPI00309704	IPI00200070	
Pancreatic alpha-amylase	IPI00133544	IPI00211904	
Parotid secretory protein	IPI00131763	IPI00209118	Also has number IPI00881015 in mouse
Polymeric immunoglobulin receptor	IPI00310059	IPI00205255	
Prolactin-inducible protein homolog	IPI00113711	IPI00198494	“Salivary proline-rich protein” in rat
Serum albumin	IPI00131695	IPI00191737	
Titin isoform N2-A	IPI00604969	IPI00959327	“titin” in rat

We focused on proteins that are members of large families because they provide opportunities to investigate both the evolutionary histories of their expansions and their sequence evolution as signatures of selection. We also culled out proteins that appear to show sex-limited expression in mouse and rat saliva (Table S6). Sex-limited expressions are defined as those in which only one sex expresses the protein or where there is a large disparity in the quantity of the protein expressed by the two sexes. Using the Quantify function of the Scaffold program to identify proteins expressed in only one sex (Table S6, part a) and manually curating the proteome data to add proteins with clearly disparate quantities between the two sexes (Table S6, part b), we identified 29 proteins in mouse saliva (36%) and nine in rat saliva (20%) with sex-limited expression. In the mouse, 24 of 29 of the sex-limited expressions were found in the male while only five were found in the female. In evaluating the data listed in Table S6a, however, we emphasize that evidence based on identification of one or a few spectra for the protein in one sample and none in the other is suspect (e.g., Isoform 2 of Myosin XVIIIa).

Twelve of the male sex-limited expressions were members of the Klk1b mouse subfamily. All but one member of this mouse-specific kallikrein subfamily (thought to be expressed genes rather than pseudogenes [30]) were expressed in male mouse saliva but were not present in female mouse saliva. Only Klk1 and Klk1b5 showed expression in both sexes and in both cases, there was substantially more expression in female saliva.

Surprisingly, only one kallikrein, Klk1 (identified in the IPI database as KLK1b3), was found in saliva of the genome rat (Table 1) and was there in roughly equal quantities in both sexes. This stands in strong contrast to reports identifying transcripts of all rat kallikrein subfamily genes (i.e., not characterized as pseudogenes) in male Sprague-Dawley rat submandibular glands [23]. For this reason, we pursued an investigation comparing the kallikrein transcripts found in submandibular glands of the genome rats and Sprague-Dawley rats and reproduced the results of [23]. We also compared the histology in the submandibular glands of both strains.

### Transcript expression and histology of submandibular glands in two strains of rat

**Figure 1:**
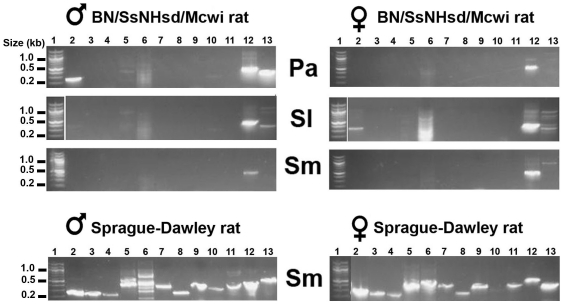
Identification of rat subfamily kallikrein transcripts in submandibular glands of the genome (BN/SsNHsd/Mcwi strain) and Sprague-Dawley rats. Submandibular gland cDNA libraries were produced by reverse transcription of total RNA. Kallikrein transcripts were amplified by PCR using primers produced from the designs published by [23] and separated on a 2% agarose gel. The samples in each gel photo are identified by the primer nomenclature of [23] with the rat gene nomenclature of [30] listed parenthetically in this figure legend. Lanes 2–11: rKLK1 (*Klk1*); rKLK2 (*Klk1c2*); rKLK3 (*Klk1c3*); rKLK4 (*Klk1c4*); rKLK6 (*Klk1c6*); rKLK7 (*Klk1c7*); rKLK8 (*Klk1c8*); rKLK9 (*Klk1c9*); rKLK10 (*Klk1c10*); rKLK12 (*Klk1c12*). Lane 1: 100 bp DNA ladder; Lane12: GAPDH cDNA control and Lane 13: beta-actin cDNA control. It should be noted that, in the case of the two Sprague-Dawley rat gel panels, there is some curvature that causes the controls on the right end to appear to have somewhat different molecular weights than in the genome rat gel panels but this is not the case.

Figure 1 shows an analysis of kallikrein subfamily transcripts found in the salivary glands of the genome and Sprague-Dawley strains of rat using the primers designed by [23] to amplify cDNAs produced by RT-PCR. The quality of the cDNA was assessed by two internal controls, GAPDH and β-actin. The genome rat males showed expression of Klk1in parotid glands, and females in the sublingual glands, but otherwise neither showed much evidence of transcription of kallikrein subfamily transcripts, with the possible exception of minor amounts of Klk1c6 and Klk1c7 in both sexes. In striking contrast to the genome strain of rat, Sprague-Dawley rats of both sexes had transcripts of all rat Klk1c family kallikreins in submandibular gland cDNA pools, as had been reported by [23] for males. This observation cannot be explained by a difference in diet [37], [38], [39], [40] as the two strains of rat used in this study were maintained on the same diet (see Materials).

Expression of most of the rodent-specific kallikrein subfamily genes has been reported to correlate with elaboration of the granulated convoluted tubular (GCT) tissue in male rodents [17], [41], [42], [43]. We wished to determine whether the lack of kallikrein subfamily gene expression in male genome rats was concomitant with a lack of GCT in this strain, so we evaluated the histology of submandibular glands in both strains and genders of rat (Figure S2). Briefly summarized, the submandibular tissue of both sexes of genome rat and both sexes of Sprague-Dawley rat shows, to a greater or lesser extent, elaboration of the GCT with typical basal nuclei and prominent secretory granules in the Sprague-Dawley rat glands compared to the genome rat glands. Females appear to have less GCT than males but the difference is not striking. The histological similarity between the two rat strains stands in contrast to the differences in kallikrein gene expression. It does not support either the notion that elaboration of GCT is sex-limited in rats nor the oft-stated assumption that expression of the subfamily (Klk1c) kallikreins in rat is a consequence of GCT elaboration in the submandibular glands of adult males. By contrast, elaboration of the tubular portion of mouse submandibular glands has been shown to occur at puberty [27], [44] and can be induced by treatment with testosterone [44]. The tubular portion of the submandibular gland of males synthesizes and secretes unique proteins [45], as well as higher levels of protease [44] and nerve growth factor (Klk1b3 and Klk1b4) [46], which appear in male mouse salivas following puberty. Our observation of sex-limited expression for all but one member of the mouse Klk1b subfamily is consistent with those earlier reports.

### Previously described mouse pheromonal proteins in the mouse and rat saliva proteomes

We were also interested in the expression in rodent salivas of members of large protein families that have been reported to have communication roles in rodents. The intensively studied ABP protein family was originally discovered as a small dimeric protein highly represented in mouse saliva and having its origin primarily in the submandibular gland [47], [48]. The completed mouse genome has recently been shown to contain 64 genes and pseudogenes for this protein family [49]. The mouse proteome we report here supports previous findings of expression of three paralogs, *Abpa27*, *Abpbg26* and *Abpbg27* in salivas of both males and females [50], [51]. Female salivas appeared to have more *a27* and more *bg26*, while values for *bg27* were about equal (Table S1). Even when we lowered the threshold criteria, we did not see the other gene products whose transcripts were previously reported [28]. We expected to find at least some *Abp* gene expression in rat saliva, given the high expression levels in mouse saliva but we did not find any in the saliva proteome of either sex of the genome rat [52]. However we did observe modest levels of at least one alpha subunit transcript in Sprague-Dawley submandibular gland (not shown).

The exocrine secretory peptide genes ESP4 and ESP8 have been shown by RT-PCR to be expressed in the submandibular gland of sexually mature BALB/c mice [53]. Our proteomics study did not detect any member of the ESP family of proteins in the salivas of the genome mouse, even though we verified the presence of the protein sequences in the mouse IPI database. However, ESPs are small proteins with molecular weights of less than 10 kDa, with many lysine and arginine residues, and thus we recognize that our MUDPIT analysis may not have been able to recognize the small peptides produced by treating them with trypsin, [53]. It is also possible that the C57BL/6 strain we studied does not express the same ESP profile as the BALB/c strain.

The third proteinaceous pheromone family that has received considerable attention is the major urinary proteins (MUPs; see [54] for a recent review). MUPs are small (18–19 kDa) barrel-shaped lipocalin proteins with a central cavity that binds small lipophilic molecules. We encountered MUP5 in male mouse saliva at 9-times the amount found in female saliva, but this was below our threshold for inclusion in Table S6 (see also Table S1). We found no MUP proteins in the salivas of rats of either sex.

One means of searching for a protein which might otherwise be expected to appear in a particular proteome analysis is to reduce the stringency of the Scaffold criteria for identification, usually starting by lowering the requirement for at least two peptide identifications to one. We tried reducing a number of stringency criteria, separately or in combination, but were unable to find any evidence for a peptide of any of the rat ABP subunits or of any ESP or MUP in the genome rat saliva. Provisionally, we must conclude that these proteins were not expressed in the genome rat. The absence of an ABP in rat saliva, coupled with the absence of expression of any of the rat-specific subfamily kallikreins, leads us to conclude that the genome rat has a very different salivary protein expression pattern than those seen in standard strains of laboratory rat.

### Gene conversion has contributed minimally to similarity among rodent kallikrein subfamily paralogs

We began our study of the evolutionary history of the mouse and rat kallikrein subfamilies by asking if gene conversion has contributed significantly to sequence identity in the mouse and rat kallikrein subfamily paralogs. Exchange of genetic information through gene conversion or ectopic recombination between tandem gene copies following duplication can also obscure divergence because changes in one of the duplicated gene copies may be erased. Our GENECONV analysis of mouse kallikrein subfamily paralogs identified three inner fragments (conversion between genes within alignment), all of which involved the *Klk1b4* paralog (*Klk1b5* and *Klk1b4*; *Klk1* and *Klk1b4*; *Klk1b1* and *Klk1b4*) and three outer fragments (conversion with genes outside alignment: *Klk1b4*, *Klk1b9*, *Klk1b22*) that were globally significant. In the case of the rat kallikrein subfamily paralogs, the analysis identified only one inner fragment (*Klk1c3* and *Klk1c7*) and two outer fragments (*Klk1c2*, *Klk1c12*) that were globally significant. We also calculated the GC content of the rodent kallikrein gene regions because sequences undergoing frequent gene conversion, either ectopic or allelic, are expected to become GC rich [55], [56]. We found that the average GC content in the two kallikrein gene regions is low, 46% in the mouse subfamily region and 49% in the rat subfamily region, compared with genes undergoing gene conversion, which have much higher GC contents [55], [57], [58]. Thus, it appears that gene conversion has made a minimal, but not nonexistent, contribution to the evolutionary history of the rodent-specific kallikrein gene families. It certainly has not been significant enough to adversely affect our analysis of recently duplicated products presented here.

### Positive selection in the evolution of mouse and rat kallikrein subfamily paralogs

**Figure 2:**
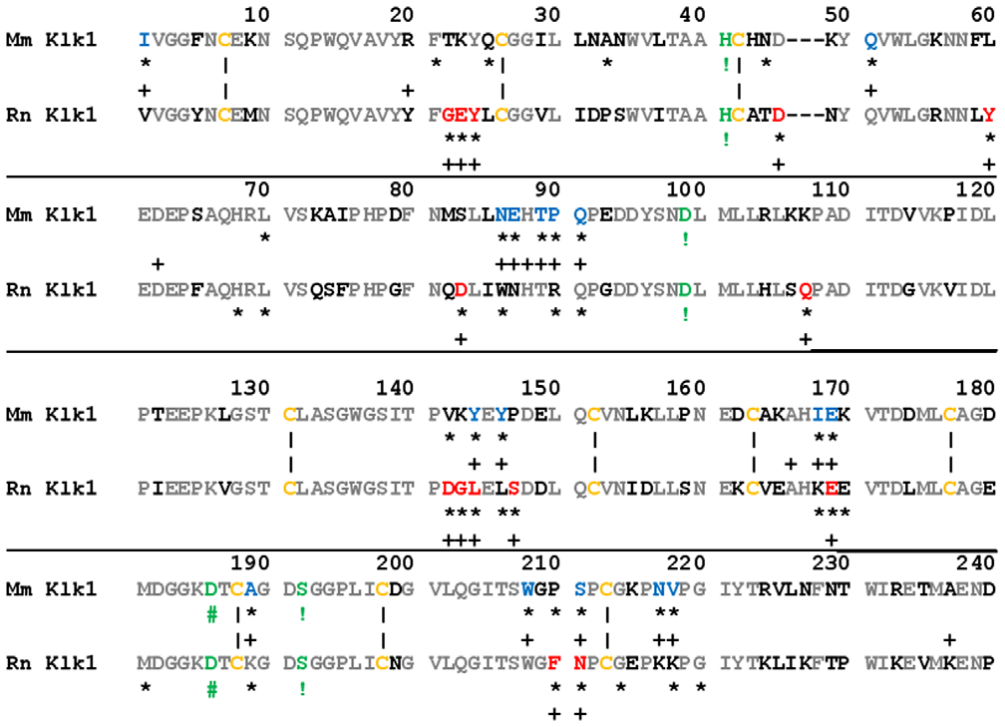
A comparison of the ω+ sites found in the mouse and rat species-specific kallikrein families, using the CODEML program (an independent analysis for each subfamily). The alignment of the basal Klk1 sequences used in this figure contains a three-residue gap so that the numbering system will be compatible with Table 2. The numbering system begins with the first amino acid residue of the cleaved proenzyme (i.e., the active form of the enzyme). This changes the numbers of the active site triad (sites indicated with ! and in green typeface) from His57 to His41, and with the three-residue gap Asp112 and Ser205, become Asp99 and Ser192. The number of the D199 that influences substrate specificity (site indicated with #) is changed to 186. Asterisks mark the sites with posterior probabilities greater than 0.9 calculated without removing GENECONV sequences. Plus signs (+) mark the sites with posterior probabilities greater than 0.9 calculated after GENECONV sequences have been removed. Amino acid residues identical between the mouse and rat Klk1 sequences are shown in gray typface, while differences are shown in black. Sites positive both with and without GENECONV sequences are identified with blue typeface in mouse and red in rat. Half-cystine residues are shown in yellow typeface and oriented to each other with vertical dashes.

Given the size of the mouse kallikrein subfamily and its striking dominance among expressions limited to the male sex, we pursued a molecular evolutionary study of those proteins. To assess the role of positive selection in the recent expansions of both the mouse and rat species-specific kallikrein subfamilies (listed with their gene coordinates in Table S7) we employed the CODEML program in the PAML package [25], [26]. Table 2 shows a summary of the CODEML results, run both with and without the paralogs potentially subjected to gene conversion, which indicate that positive selection has acted at many sites, designated ω+ sites, on both sets of paralogs. The gene trees used in CODEML analysis are shown in Supp. Figure S3. The ω+ sites are mapped on sequences of KLK1 in mouse and rat shown in Fig. 2 (aligned FASTA sequences of all paralogs are presented in Figure S4). There is apparent similarity in the regions of these sequences where positively selected sites map. We also ran CODEML without the paralogs potentially subjected to gene conversion (six of fourteen mouse sequences were removed and four of ten rat sequences were removed, Table 2). While it is still evident that positive selection acted on the remaining sequences, the number of selected sites was reduced in the second analysis (23 to 21 in mouse and 27 to 14 in rat).

**Table 2:** Comparison of selection test on mouse and rat kallikrein subfamily genes with and without the paralogs identified as possibly undergoing gene conversion.

Rodent species	Gene subfamily	Ratio of *dN/dS* (%Codons) a	p-Value: All species b	Codon Sites (ω+ sites) under Selection c
*Mus musculus*	*Klk1b*	3.5 (15.8%)	p<0.001	**I1**, **F21**, **Q25**, **A33**, **N44**, **Q48**, **L67**, **N83**, **E84**, **T86**, **P87**, **Q88**, **V139**, **Y141**, **Y143**, **I165**, **E166**, **A186**, **W205**, **P207**, **S208**, **N214**, **V215**
*Rattus norvegicus*	*Klk1c*	2.77 (17.6%)	p<0.001	**G22**, **E23**, Y24, D45, **Y60**, **H68**, L70, **D83**, W86, R90, **Q91**, **Q107**, **D142**, **G143**, **L144**, **L146**, **S147**, K168, **E169**, E170, M181, **K189**, F210, **N211**,G214, **K218**, G220
Removing GENCONV sequences				

Rodent species	Gene subfamily	Ratio of *dN/dS* (%Codons) a	p-Value: All Species b	Codon Sites (ω+ sites) under Selection c
*Mus musculus* Klk1, b1, b4, b5, b9 and b22 removed	*Klk1b*	5.73 (11.2%)	p<0.001	**I1**, R20, **K51**, **D62**, **R86**, **K87**, H88, **I89**, **R90**, **F91**, **F144**, **F146**, A166, **I168**, **E169**, **K189**, **W208**, **T211**, **D217**, **M218**, **A237**
*Rattus norvegicus* c2, c3, c7 and c12 removed	*Klk1c*	3.5 (12.7%)	p<0.001	**G22**, **E23**, **Y24**, **D45**, **Y60**, **D83**, Q107, **D142**, **G143**, **L144**, **S147**, **E169**, **F210**, **N211**

a The *dN/dS* ratio of the class of codons under positive selection is given with the percentage of codon sites predicted to be in that class.

b The p-value rejecting the model of neutral evolution (M8A) over that of selection (M8) is given.

c Sites with posterior probabilities greater than 0.9 are indicated in regular typeface; p>0.95 indicated in bold typeface and p>0.99 indicated in bold, underlined typeface.

**Figure 3:**
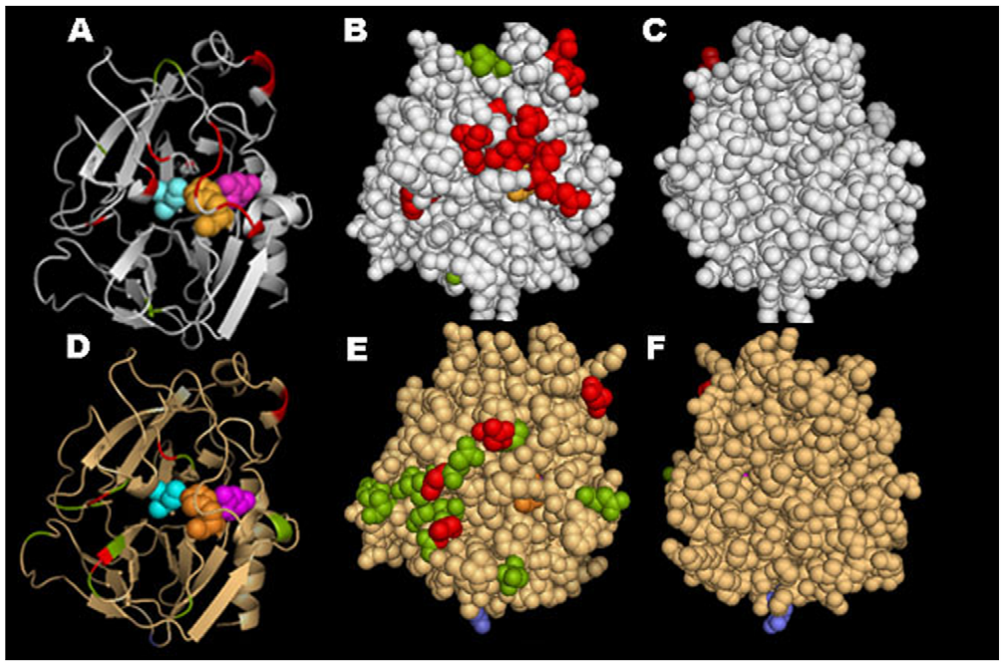
Positive selection at sites on molecular models of mouse and rat species-specific kallikreins. Because both rodent Klk1 kallikreins mapped on the d1gvza model, that was used with each Klk1 sequence to produce the mouse and rat molecular models with PyMol. Residues in red are selected with a BEB posterior probability >99%; those in green with a BEB posterior probability >95%; and those in blue with a BEB posterior probability >90%. Panels A and D: Cartoon model of mouse and rat subfamily kallikreins, respectively, with α-helices shown as spiral tapes and β-sheets shown as flat arrows. The active site triad is shown in spheres colored light orange (His41), pink (Asp96) and light blue (Ser189). The two Greek key motifs, consisting of β-sheets can be seen above and below the active site residues. Solid models of mouse (Panels B and C) and rat (Panels E and F) show the fronts (B and E, where B is the same orientation as A, and E is the same orientation as D) and backs (C and F) of the two species-specific kallikreins. Essentially all the sites under selection map to the front surfaces of both structures.

To determine whether the positively-selected sites in the Klk1 sequences of the two rodent kallikrein subfamilies correspond to similar domains in their three-dimensional structures, we modeled them using the PHYRE threading program (Table 3). The resulting models were visualized using PYMOL (Fig. 3). Taking the more conservative approach, we only mapped those sites identified without the putative paralogs subjected to gene conversion. The overall conclusion in comparing the mouse and rat models is that essentially all of the sites under positive selection map on the surface of the same face of both proteins. The open cartoon figures (Fig. 3 Panels A and D) include identification of the active site triad of amino acid residues, none of which show the effects of positive selection in either mouse or rat. It is possible that the surface with the positively selected sites interacts with another molecule(s).

**Table 3:** Mouse and Rat *Klk1* Genes Used to Produce Molecular Models.

Rodent	Accession Number	Chromosomal Location (strand)	Threaded Structure a (results) b
*Mus musculus*	NP_034769	chr7:51222157–51226640 (+)	d1gvza (100%; 237; 54%)
*Rattus norvegicus*	NP_001185899	chr1:79194543–79202526 (+)	d1gvza (100%; 237; 54%)

a The cleaved proenzyme sequence (i.e., the active enzyme sequence) was threaded for this study.

b Data consist of structural model, % confidence, length, and % identity.

## Discussion

The major advantage of modern proteomics analytical methods using tandem mass spectrometry (MS/MS) lies in their ability to provide comparative data on many proteins in a biological fluid or tissue extract. The LC-LC-MS/MS MUDPIT technique that we applied to the study of rodent salivary proteomes reported here should provide a snapshot of the most highly represented proteins in a single sample. However, MS/MS methods also suffer from the limitation that the specific database search engine thresholds required to identify peptides and thus proteins with the most reliability will ignore single spectra and/or weak spectra that are otherwise actual signals of the presence of proteins in very small quantities in the sample. In some cases it might be appropriate to reduce the stringency of the Scaffold criteria, either by lowering the requirement for at least two peptide identifications to one and/or by changing other search engine threshold settings. However, this should only be done to provisionally detect signals that can be verified with another line of experimentation, rather than accepting the result itself as evidence of detection of a particular protein.

Saliva is a complex mixture of several highly abundant proteins (e.g., alpha-amylase), as well as many glycosylated proteins and some proteins rich in proline and/or other amino acids. Abundant proteins tend to mask lower abundance proteins in MUDPIT analysis and so steps were taken to limit this problem. We used two techniques to control highly abundant protein problems: 1) Dynamic exclusion settings during the acquisition, and 2) controlling the total amount of protein. For dynamic exclusion the instrument is set to record a mass spectrum (MS), and then to take the top most intense ions (usually seven) and fragment them (MS/MS) one-by-one. The instrument parameters are set to acquire these MS/MS spectra a certain number of times (1–3) during a certain amount of time, usually 30 seconds. If that criterion is met then the precursor mass (mass observed during the MS) is added to an exclusion list and its MS/MS data is not acquired by the instrument for a specified period of time, usually 45 sec-2 min. While the dynamic exclusion settings reduce the instrument focus on abundant peptides, we may still observe them at higher number because the column is “overloaded” with them and they can continue to elute from the column for long periods. However, less abundant proteins are still observed because of the dynamic exclusion settings. We note, however, that even when dynamic exclusion is used, some low-abundance peptides will be missed because, when one abundant peptide is sampled repeatedly and excluded, other variants of the same peptide with slightly different masses can still predominate in the same sampling window and still mask other low-abundance peptides [59].

The Proteomics Core Facility of the University of Arizona has experimentally determined that around 30 µg of protein is the optimal sample amount to be loaded onto their 12-step MUDPIT to observe the greatest number of lower abundance proteins in the presence of the higher abundance ones. A larger sample will bring more proteins to the highly abundant/overloaded state described above and a smaller sample will result in fewer protein identifications.

Our MUDPIT results yielded roughly twice the number of proteins in stimulated mouse salivas (81) as in stimulated rat salivas (46). While some of the difference may be attributed to single male and female salivas having been analyzed in rat whereas we analyzed three of each sex in mice, the results do not suggest that as the only explanation. In fact, when proteins with two or fewer identifications in the three datasets of both sexes are removed from the mouse list (not shown), the number of proteins identified falls from 81 to 58, which is still 26% more proteins in the mouse than in the rat results. While this might be simply explained by the larger number of proteins (∼50% more) in the mouse IPI database than in the rat database, it may not be the only explanation. We suspect that lack of expression of any of the nine rat Klk1c subfamily kallikreins, along with other proteins we expected to find (e.g., at least one alpha and one beta-gamma subunit encoded by the six rat *Abp* genes) can account for about half the difference, since transcripts for these proteins have been found in salivary glands of other rat strains and, indeed we have repeated those results here. Moreover, the groups of sex-limited expressions for both sexes of the two rodents have similar numbers when the mouse subfamily kallikreins are removed from the male mouse sex-limited expression group. It seems clear from the results we report here, that the genome rat has a substantially different expression pattern, particularly in terms of the sex-limited expression of rat Klk1c family kallikreins and ABPs, which may be explained, at least in part, by an apparently lower number of secretory granules in the GCT of their submandibular glands, than is found in other rat strains such as the Sprague-Dawley strain of laboratory rat. The striking differences we have observed in the saliva proteomes of mice and rats reinforces the anecdotal conclusion that rats are more than just big mice.

Mammals share a set of serine proteases called kallikreins, which have been extensively characterized and have been the subjects of recent reviews [18], [20]. In addition, some mammal species, including mouse, rat, horse and possibly shrew have elaborated subfamilies of kallikreins specific to them [18], [20], [60]. Previous studies by others identified RNA [23], [61], [62], [63], [64], [65], [66], [67], [68], [69] and protein [70], [71], [72], [73] of kallikrein subfamily genes in rodent submandibular glands. Much of this previous work, however, focused on kallikrein expression in the submandibular glands of males only. In the few cases in which both sexes were investigated, mice show a clear sexual dimorphism with males expressing more RNA of the mouse-specific subfamily members than females [63], [64], [67], [68]. Studies of the rat-specific family expression have not been so clear. Older literature suggests similar kallikrein enzyme activity between male and female Sprague-Dawley rats [72], while newer literature suggests that males express more RNA and protein for at least some family members [66], [73] and emphasizes the influence of androgen on kallikrein gene expression [61], [62], [73]. The functional purpose(s) of these species-specific kallikrein families are unknown, due in part at least because interest in them waned when it was discovered that there were no human counterparts [20]. Speculation concerning the function(s) of the independently-expanded families of salivary kallikreins is based on two studies showing that Klk1 and Klk6 increase cell migration in vitro [74], [75]. Only Klk1 is directly related to the mouse subfamily b kallikreins as the basal member of that recently expanded family. No studies have specifically examined the function(s) of the mouse- and rat-specific subfamily members (i.e., the Klk1b kallikreins of the mouse and the Klk1c kallikreins of the rat). Other work suggests that human KLK4, a kallikrein that appears in saliva and is expressed in several tissues, is important in tooth mineralization [76]. Other kallikrein functions include skin shedding and lysis of the copulatory plug [76].

We feel that the time is right to reopen the case of the function(s) of the Klk1b and Klk1c kallikreins encoded by genes that have expanded very recently in the mouse and rat genomes, respectively. Although there is evidence that other kallikreins, including Klk1 basal to the mouse subfamily expansion may play a role in wound healing, no direct evidence for that function or any other has been produced. We question whether any of these specific kallikreins are involved in wound healing and, to that end, we undertook a molecular evolutionary study of the two rodent kallikrein subfamilies. The mouse and rat subfamilies expanded independently since their last common ancestor [19] which narrows the time frame of these expansions to about 12 MYR [77]. Given the strong evidence that mouse subfamily b kallikreins are expressed in an almost wholly sex-limited fashion (only Klk1b5 is not), we pursued the question of what role positive selection might have played in the independent expansion in this species and, for comparative purposes, we also analyzed the rat subfamily kallikreins. Before doing so, however, we reconsidered the role that concerted evolution played in the evolution of these two subfamilies.

We did not find evidence of pervasive gene conversion in the mouse-specific and rat-specific kallikrein families, either by application of GENECONV or by estimation of GC content. Employing the CODEML program in the PAML suite of programs [25], [26], we asked what role positive selection played in that evolutionary history (see [76] for a study involving a different approach). We found strong evidence of positive selection in each subfamily, involving many amino acid sites in both mouse- and rat-specific kallikrein sequences. This was true whether the kallikrein sequences identified by the GENECONV analysis were included or not, although the number of sites identified as under positive selection was diminished in both subfamilies when sequences were removed, as would be expected. Most importantly, the positive selection occurred in a pattern that strongly suggests that rapid evolution must have involved similar adaptations in both families. In summary, our results show that the amino acid sites under positive selection are essentially all on the surface of the proteins, rather than affecting interior sites such as the active site triad. It is particularly striking that these rapidly evolving sites appear almost entirely on one face of each protein and that those are the same face on paralogs of both subfamilies, suggesting that these two subfamilies have experienced convergent evolution.

We suggest that the evolutionary results we report here call for a reconsideration of the function of salivary kallikreins in rodents. Although we do not dispute the notion that one function may be wound healing, the logic that males need this and females do not seems to us naïve. Such a simple explanation for independent expansion and concomitant convergent evolution of the two species-specific kallikrein subfamilies appears to be inadequate for several reasons. There are a number of studies of mouse behavior that show that female mice will defend their nest and offspring from foreign males or intruding females [78], [79] and in-so-doing, they must occasionally be wounded. Wounds in both sexes probably also arise occasionally from contacts with predators from which they, the prey, escaped.

Beyond speculation, the observations we report here also do not square with the notion that wound healing is the sole function of these unique kallikreins. We found that both adult male and female genome rats show about equal elaboration of GCT in their submandibular glands but little or no detectable Klk1c subfamily kallikrein expression, either in terms of submandibular transcripts or proteins in saliva. This rather surprising finding suggests that this strain of rat does not rely on the subfamily kallikreins in saliva for wound healing, although it is difficult to imagine that the males do not show aggressive behavior towards each other in competition for females. If a salivary kallikrein is in fact required for wound healing in either sex of genome rat, then it must be Klk1, consistent with the observations of [74], which is the only kallikrein expressed and that in both sexes. By contrast, both adult male and female Sprague-Dawley rats express all the Klk1c subfamily kallikrein transcripts in their submandibular glands, consistent with a relatively high level of GCT in the glands in both sexes. Our observations on these two rat strains calls into question: 1) the absolute reliance on increased levels of circulating testosterone for submandibular GCT elaboration, at least in rats; and 2) promotion of wound healing alone as the function of these rodent subfamily kallikreins.

It also seems to us unlikely that wound healing would require as many as thirteen different paralogs be expressed in male mouse saliva (ten in rat saliva) although we cannot rule that out. On the other hand, our observations of rapid evolution of the paralogs in each subfamily suggests that we should consider other possible functions such as immunity, reproduction, chemosensation and toxin metabolism, defining the groups of mammalian proteins most often involved in rapid evolution (see Introduction). Moreover, we suggest that the apparent convergent evolution, in terms of the concentration of positively selected sites on the same surface of both families of proteins, suggests that they interact in a recognition context with other molecules. Candidates for interaction partners could be receptors such as the VNO receptors if communication is involved and/or surfaces of other proteins, such as ABPs that also have positively selected sites on one face of the protein [80], if the two are required to function together. We realize that these suggestions are speculative and tenuous at best, however, perhaps they will generate new interest in the functions of these rodent-specific families of kallikreins. We propose that the evident sex-limited expression in mice at least, coupled with their rapid evolution may be clues to an as-yet-undetermined interaction between the sexes. An intriguing possibility is that these subfamily kallikreins function to inactivate and/or damage proteins with a signaling function produced by competing animals.

The difference we report here in the expression of *Klk1c* genes between the genome rat strain and the Sprague-Dawley strain is intriguing. We feel it is most likely that the explanation is some form of genetic drift, rather than natural selection. The two rat strains in question were derived from independent samplings of the *Rattus norvegicus* gene pool in the last century and the progeny were maintained in captivity, and therefore it seems unlikely to us that selection, natural or artificial, was involved in these breeding programs. The first inbred rat strain was PA, derived from albino stocks of *R. norvegicus* in the Wistar Institute, Philadelphia, in 1909 [81], [82]. Apparently both the Wistar and Sprague-Dawley albino strains arose from that strain, although the Wistar strain also has genetic material from hooded rats. The genome rat strain has a complex history, apparently very different from that of the commonly used albino strains. It was obtained for the rat genome project as a Harlan stock but was found to contain unwanted genetic diversity and so was inbred another 13 generations before being used as a source of DNA for sequencing the rat genome [1]. It has a brown agouti coat rather than an albino one suggesting that its origin was a different sample of the the *R. norvegicus* gene pool than that used to create the albino inbred strains. Thus the more likely explanation of the differences in *Klk1c* gene expression in the two strains we studied here is founder effect, that is to say independent samplings of the *R. norvegicus* gene pool. Our proposal is supported by the observation [83] that the strain used for sequencing the rat genome consistently possesses a genetic divergence most distant from all other strains [83].

## Supporting Information

Figure S1 Male and female mouse scatterplots of Sequest identifications vs. X! Tandem identifications with the custom criteria set in the Scaffold software analysis (see Methods). The vertical dashed line represents the 2.5 setting in Sequest, corresponding to peptides with 2 charges, the most common spectrum encountered. The horizontal dashed line represents the X! Tandem setting of 2 (actually 10^−2^), corresponding to a probability of 1% or less of a mis-identification. Those identifications mapping in the upper right hand quadrant of the dashed line intersection meet the custom criteria. The solid line demarcates the threshold of a combined 95% probability of a correct identification.
(TIF)

Figure S2 Submandibular gland histology of two laboratory strains of *Rattus norvegicus*: RN is the genome rat, BN/SsNHsd/Mcwi strain, and SD is the Sprague Dawley strain. Tissues were fixed in 10% neutral buffered formalin for 24 hours before embedding in paraffin. Serial sections of 4.5 µ were cut and stained with hematoxylin and eosin.
(TIF)

Figure S3 Phylogenetic trees of the mouse and rat species-specific kallikrein subfamilies used in CODEML analysis. The cleaved pro-kallikrein sequences representing active enzymes were aligned using CLUSTALX [31], [32] and saved as nexus files. Phylogenetic trees were constructed from the alignments using the program PAUP* (neighbor-joining distance parameters with Jukes-Cantor correction) [33] and these were displayed in TreeView [34]. Bootstrap values were calculated with 1000 replications.
(TIF)

Figure S4 FASTA alignments of the mouse and rat species-specific kallikrein subfamilies were made with DNAsis Max (Hitachi) and the alignments saved as a text file.
(FA) Click here for additional data file.

Table S1 Protein report for the genome mouse (C57BL/6 strain).
(XLS)

Table S2 Peptide report for the genome mouse (C57BL/6 strain).
(XLS)

Table S3 Mouse sample report.
(XLSX)

Table S4 Protein report for the genome rat (BN/SsNHsd/Mcwi strain).
(XLS)

Table S5 Peptide report for the genome rat (BN/SsNHsd/Mcwi strain).
(XLS)

Table S6 Sex-limited expressions in rodent salivas.
(XLS)

Table S7 Kallikrein subfamily gene coordinates.
(XLS)
