# Supplementary figures and images for: Positive Selection Shaped the Convergent Evolution of Independently Expanded Kallikrein Subfamilies Expressed in Mouse and Rat Saliva Proteomes
Source: PLoS One. 2011 Jun 14;6(6):e20979. doi: 10.1371/journal.pone.0020979 (PMC3114847; doi:10.1371/journal.pone.0020979)

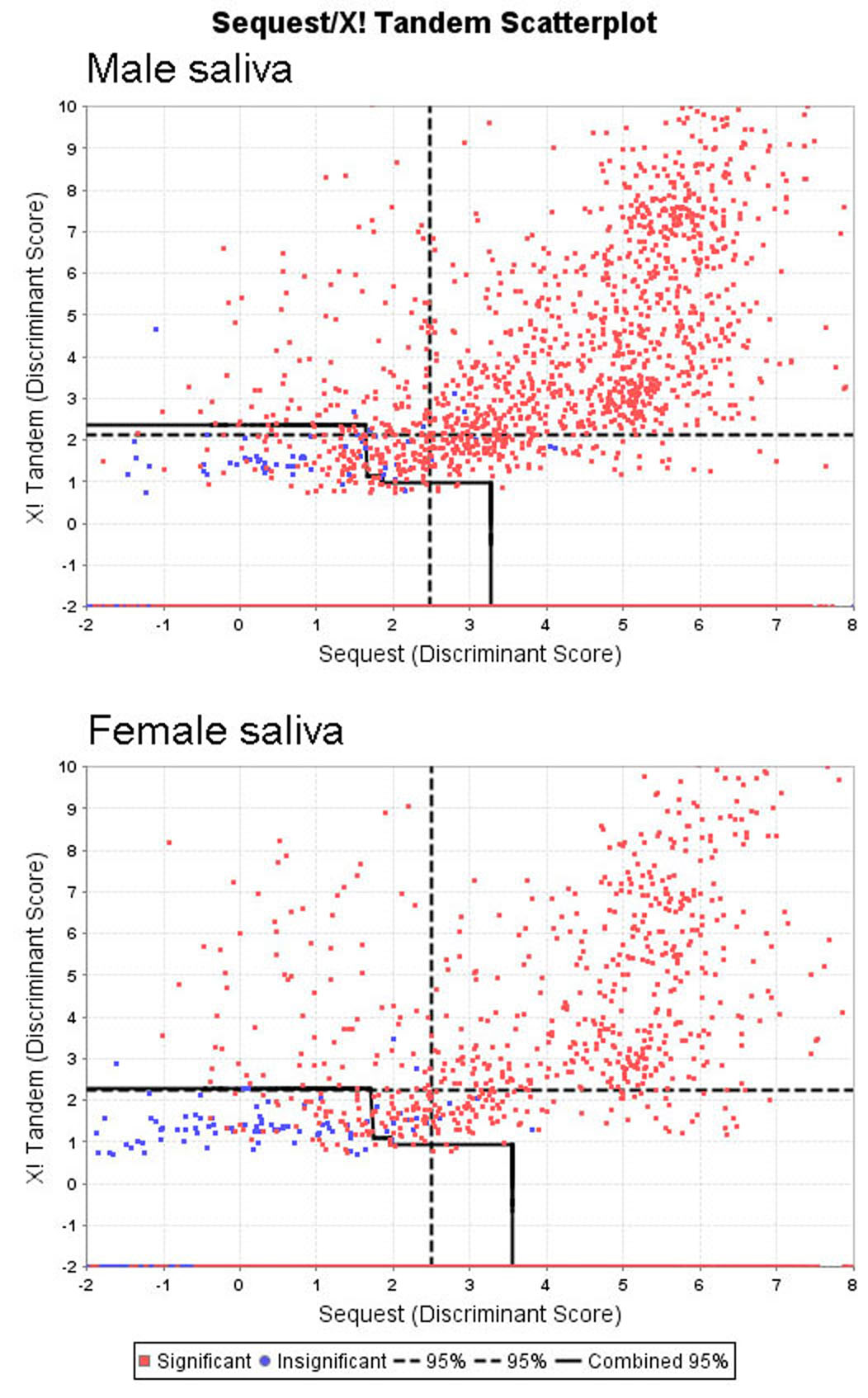

Supplement: Figure S1 — Male and female mouse scatterplots of Sequest identifications vs. X! Tandem identifications with the custom criteria set in the Scaffold software analysis (see Methods). The vertical dashed line represents the 2.5 setting in Sequest, corresponding to peptides with 2 charges, the most common spectrum encountered. The horizontal dashed line represents the X! Tandem setting of 2 (actually 10−2), corresponding to a probability of 1% or less of a mis-identification. Those identifications mapping in the upper right hand quadrant of the dashed line intersection meet the custom criteria. The solid line demarcates the threshold of a combined 95% probability of a correct identification. (TIF) [file pone.0020979.s001.tif]

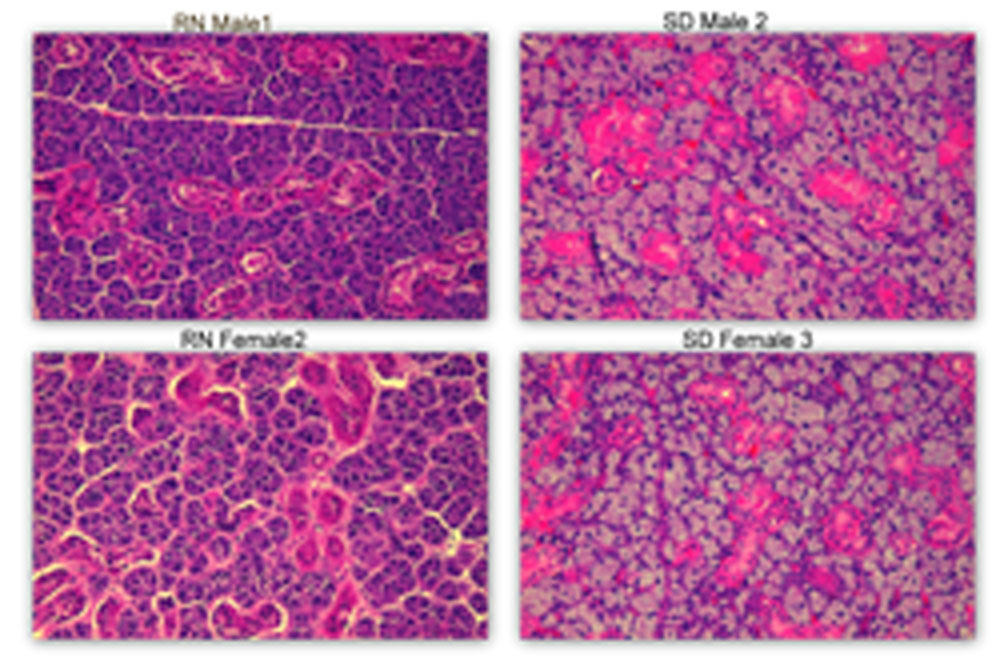

Supplement: Figure S2 — Submandibular gland histology of two laboratory strains of Rattus norvegicus: RN is the genome rat, BN/SsNHsd/Mcwi strain, and SD is the Sprague Dawley strain. Tissues were fixed in 10% neutral buffered formalin for 24 hours before embedding in paraffin. Serial sections of 4.5 µ were cut and stained with hematoxylin and eosin. (TIF) [file pone.0020979.s002.tif]

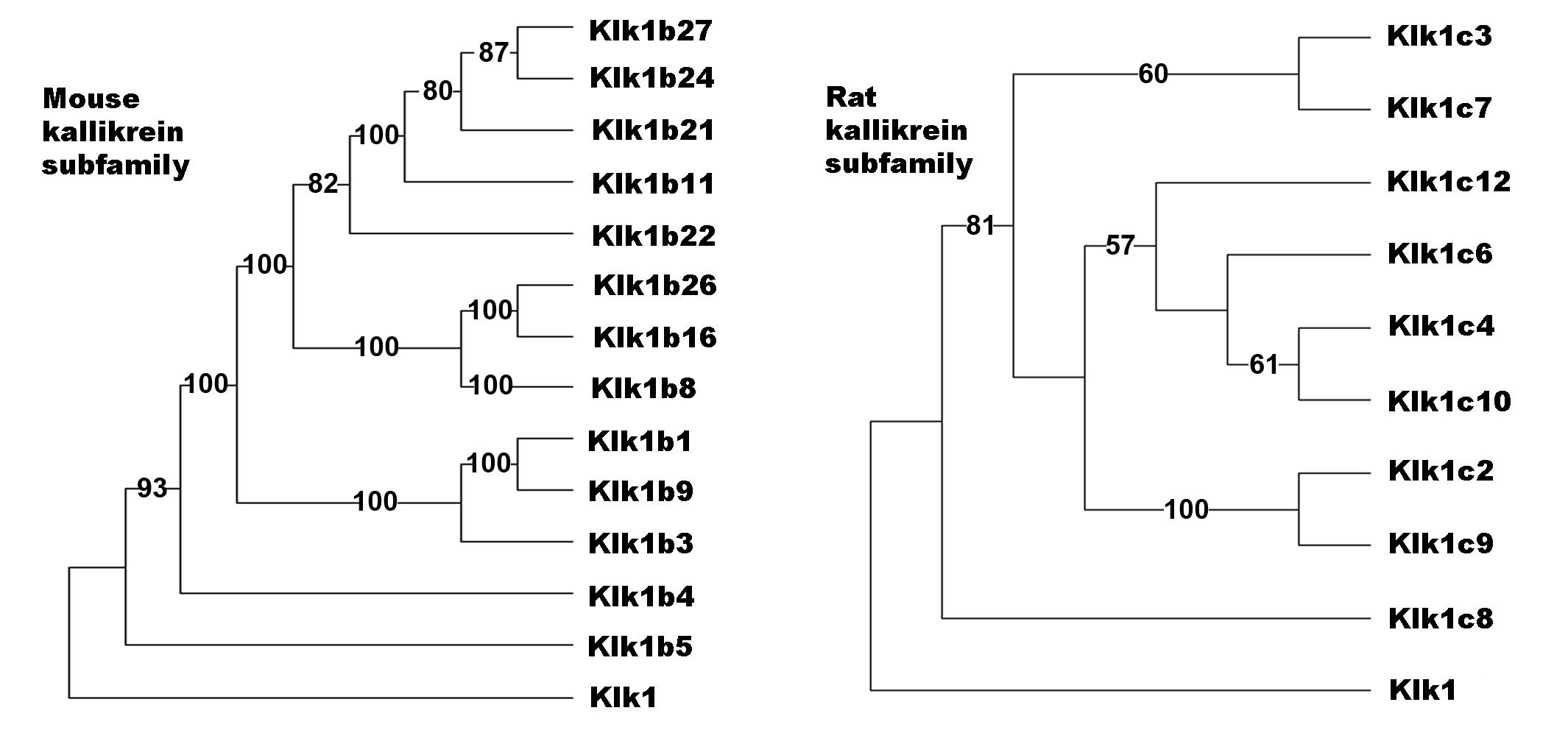

Supplement: Figure S3 — Phylogenetic trees of the mouse and rat species-specific kallikrein subfamilies used in CODEML analysis. The cleaved pro-kallikrein sequences representing active enzymes were aligned using CLUSTALX [31], [32] and saved as nexus files. Phylogenetic trees were constructed from the alignments using the program PAUP* (neighbor-joining distance parameters with Jukes-Cantor correction) [33] and these were displayed in TreeView [34]. Bootstrap values were calculated with 1000 replications. (TIF) [file pone.0020979.s003.tif]
